# Minimally Invasive Surgery for Vesicocervical Fistula Following Vacuum-Assisted Delivery with History of Cesarean Section

**DOI:** 10.3390/reports8020046

**Published:** 2025-04-11

**Authors:** Philipp Meyer-Wilmes, Tomáš Kupec, Julia Wittenborn, Elmar Stickeler, Laila Najjari

**Affiliations:** Department of Obstetrics and Gynecology, University Hospital of RWTH Aachen, 52074 Aachen, Germany

**Keywords:** vesicocervical fistula, minimally invasive surgery, postpartum repair

## Abstract

**Background and Clinical Significance**: A vesicocervical fistula is an abnormal connection between the urinary tract and the cervix. With the increased prevalence of cesarean sections in recent years, the incidence of vesicocervical fistulas has also increased. The objective of this study was to evaluate the available evidence regarding the laparoscopic approach and to present a case study of a patient who underwent minimally invasive repair of a vesicocervical fistula after vacuum delivery. **Case Presentation**: A 32-year-old mother was admitted to our center with symptoms of urine leakage through the cervix uteri 5 days after vacuum-assisted delivery. In particular, the patient had recently undergone a vacuum-assisted delivery and cesarean section. A positive methylene blue staining test and transvaginal ultrasonography demonstrated an anatomical connection between the bladder and the cervix. Surgical repair of the vesicocervical fistula was performed under general anesthesia and a Foley catheter was inserted for 7 days. Urodynamic studies conducted seven days postoperative and six weeks post-surgery showed normal bladder function and capacity. **Conclusions**: Early detection and surgical correction of vesicocervical fistulas using a minimally invasive approach is crucial for the treatment of this condition. This case report emphasizes the importance of considering vesicocervical fistulas, particularly in patients with a history of cesarean section who have undergone vacuum-assisted delivery.

## 1. Introduction and Clinical Significance

Vesicocervical fistula is a pathological condition in which a direct communication pathway is present between the urinary bladder and the cervix. It represents a significant postsurgical complication that can severely impact the quality of life of affected women. The clinical manifestations include urinary incontinence, amenorrhea, and menouria [1]. Cesarean section is becoming more common, but there is a lack of scientific evidence and no standard practice for the management of these problems. Laparoscopic repair of vesicocervical fistula is a minimally invasive surgical technique that has emerged as an effective and safe treatment option for this clinical issue [1,2]. The laparoscopic approach offers several advantages over traditional open surgery, including less postoperative pain, shorter hospital stay, quicker recovery, and reduced risk of complications [3]. This case report aimed to demonstrate the technical feasibility, safety, and outcomes of laparoscopic repair for vesicocervical fistula, contributing to the evolving best practices in minimally invasive gynecological surgery. Understanding advances in the management of complex vesicocervical fistulas following vacuum delivery is crucial for healthcare professionals to ensure optimal care for postpartum women. In the following case, we illustrate the complexities of managing vesicocervial fistula following vacuum delivery after cesarean section, with a focus on minimally invasive repair.

## 2. Case Presentation

**Figure 1:**
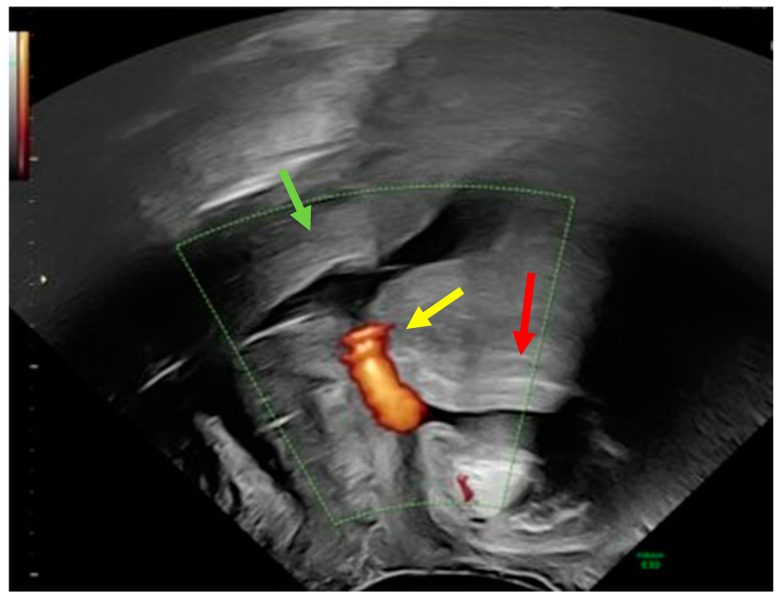
Transvaginal ultrasound using power Doppler demonstrating communication (yellow arrow) between the uterine cervix (green arrow) and bladder (red arrow).

A 32-year-old postnatal mother was referred to our tertiary university center complaining of urine leakage via the cervix uteri after vacuum-assisted delivery with impending fetal asphyxia five days prior to referral. In the past, the patient had delivered a child by cesarean section and another child by vacuum-assisted delivery. Laboratory tests did not reveal any signs of anemia or infection. First, methylene blue was injected into the bladder through a urinary catheter. Blue urine was observed when the speculum was inserted into the vagina, indicating the presence of vesicouterine fistula. In addition, abnormal communication was suspected, as transvaginal ultrasound showed a bladder wall in close contact with the anterior surface of the uterus with no demarcation line (Figure 1). 

**Figure 2:**
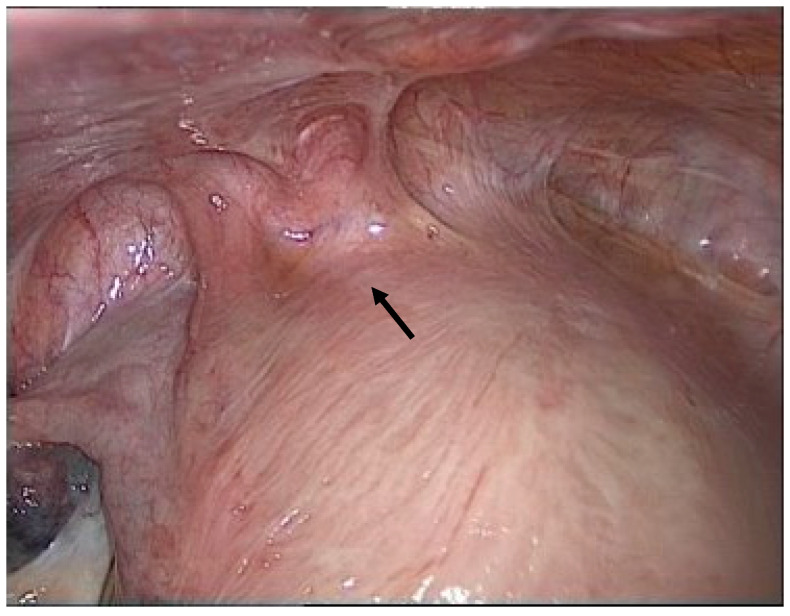
Adhesion between the bladder and uterus (black arrow).

Due to the high symptom burden, the patient underwent surgery under general anesthesia via a minimally invasive laparoscopic approach. First, significant adhesions were observed between the urinary bladder and uterine body (Figure 2).

**Figure 3:**
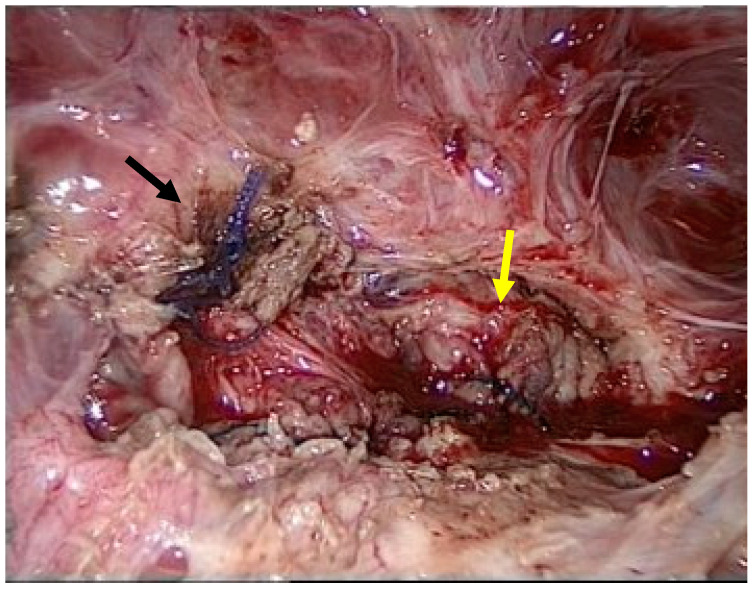
Multiple layers of sutures of the vesico urinary (black arrow) and uterus (yellow arrow).

Adhesiolysis revealed a fistula between the urinary bladder and the uterus. The presence of sutures from a previous caesarean section most likely caused this abnormal communication. The abnormal tract between the two organs was removed using partial cystectomy, cystography, and partial myomectomy. Reconstruction was performed using a multilayer technique (Figure 3).

**Figure 4:**
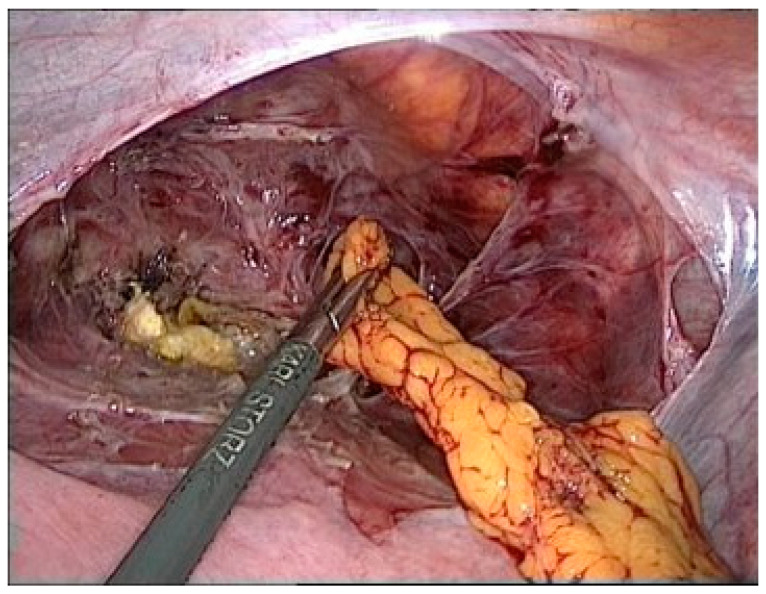
Omentum plastic.

An Omentum flap was placed between the uterine body and bladder. The flap was carefully sutured to provide additional support and to promote healing (Figure 4).

The postoperative period was uncomplicated, and the urinary catheter was removed after continent cystogram examination. The patient reported no urinary leakage or discomfort during daily activities. Urodynamic studies conducted six weeks post-surgery showed normal bladder function and capacity. We present a rare case of successful laparoscopic repair of a vesicocervical fistula after vacuum-assisted delivery, which was a long-term morbidity secondary to cesarean section.

## 3. Discussion

Uncommon complications such as vesicocervical fistulas typically occur following previous lower-segment cesarean sections or difficult instrumental vaginal births. The increase in cesarean sections over the last few decades has also had an impact on the incidence of late complications, such as vesicouterine fistulas. Common causes include inadequate and prolonged labor, previous lower-segment cesarean section, difficult vaginal delivery, trauma, and abnormal placental insertion [4].

Accurate clinical diagnosis can be difficult because of the wide range of symptoms and variability among patients. A vesicocervical fistula is a distressing condition characterized by cyclic hematuria (menouria), secondary amenorrhea, urinary continence, and secondary infertility. The initial step in identifying a genitourinary fistula involves a detailed physical examination, which can be used to visualize the fistulous tract, including transvaginal ultrasound, intravenous urography, hysteroscopy, cystoscopy, and pelvic magnetic resonance imaging. Transvaginal ultrasound, particularly color Doppler, is a less expensive and less invasive diagnostic test that can quickly and accurately identify fistulas. It can visualize fistulas as a jet phenomenon originating in the bladder and traveling to the uterus. However, MRI offers detailed images of the fistula tract and surrounding tissues, which are crucial for choosing between conservative and surgical treatment [5].

Complications from vesicouterine fistulas may arise shortly or several years after surgery. Choosing the correct treatment depends on the occurrence of these complications appear [6]. If the fistula is diagnosed early in the postoperative period with minimal symptoms, a conservative approach with prolonged use of a urinary catheter or the application of an analog of luteinizing hormone-releasing hormone for six months may be considered [7]. However, conservative treatment is usually ineffective in cases in which the fistula develops late after surgery or when there are significant symptoms. In such cases, abnormal communication between the uterus and bladder may result from severe inflammation and fibrosis. Similar to our case, an unnatural shunt was provoked during vacuum-assisted delivery. An explanation for the vesicocervical shunt may be the presence of a tissue scar, dehiscence of the uterine scar, or injury to the bladder wall at the vesicocervical interface after cesarean section. Shearing forces (e.g., vacuum-assisted delivery) on the lower uterine segment thinned by contractions can cause this acute defect. In such cases, surgical intervention is required to remove the fistula and repair the urinary bladder and the uterine cavity.

The type of surgery depends on the complex nature of the case and may be performed using laparoscopic, robotic, cystoscopic, hysteroscopic, or open approaches. Over the last two decades, the transabdominal approach has been the predominant method of repair, accounting for 67.2% of cases [2]. Minimally invasive surgery such as laparoscopic repair is a viable option for surgeons experienced in laparoscopic suturing techniques to provide optimal care. This technique is a more comfortable and efficient alternative to the traditional open surgery. It involves smaller incisions, less pain, and a faster recovery. Despite its minimally invasive nature, it achieves outcomes similar to those of open surgery [8]. For example, laparoscopic repair of vesicocervical fistulas can be performed without routine hysterectomy to preserve the uterus [9]. This approach involves excision of the fistula tract through intentional cystotomy by careful dissection of the retrovesical space between the bladder and the uterus. Furthermore, catheterization of the fistula tract during laparoscopy is beneficial for its localization. Either a transvesical or extravesical approach can be used for specific repairs. The choice between these two techniques is often based on the surgeon’s skills, comfort, and ability. The most important aspects of repair are adequate dissection and tight sutures using multiple layers of uterus and bladder. Laparoscopic repair is effective in treating vesicouterine fistulas, with successful closure of the fistula tract and resolution of the symptoms [1,2,3]. Recently, the robotic approach has been shown to be associated with improved early and long-term postoperative outcomes due to better visualization of the structures and the presence of multiple degrees of freedom, which are well-known when using robotic platforms [8]. However, the robotically assisted method must be viewed cautiously because of the lack of data and a recently published case of failure [10].

In addition to demonstrating the successful application of minimally invasive laparoscopic surgery for repairing a complex vesicocervical, this report has several limitations, including reporting bias and the fact that this was a single case report with short-term follow-up. Further research and case studies are needed to establish standardized protocols for the management of vesicocervical fistulas.

## 4. Conclusions

In conclusion, the successful management of this case highlights the effectiveness of laparoscopic repair in addressing vesicocervical fistulas. Prompt diagnosis and surgical intervention using a minimally invasive approach significantly improves patient outcomes. This case report emphasizes the importance of considering vesicocervical fistulas, particularly in patients with a history of cesarean section.

## Data Availability

The original data presented in this study are included in the article. Further inquiries can be directed to the corresponding author.
